# Rape survivors’ experience in Tigray: a qualitative study

**DOI:** 10.1186/s12905-023-02502-0

**Published:** 2023-07-13

**Authors:** Mengistu Welday Gebremichael, Birhane Gebremariam, Mengistu Mitiku, Znabu Hadush, Bisrat Tesfay, Alemseged Gerezgiher, Muauz Gidey Alemu

**Affiliations:** 1 College of Health Sciences, Mekelle University, P.O.Box: 1871, Mekelle City, Tigray, Ethiopia; 2 Tigray Institute of Policy Studies, Mekelle City, Tigray, Ethiopia; 3 Institute of Population Studies, Mekelle University, Mekelle City, Tigray, Ethiopia

**Keywords:** War, Rape, Sexual abuse, Survivors, Violence, Tigray, Ethiopia

## Abstract

**Introduction:**

As consequences of war, women and girls are the most likely segment of society to be impacted by violence. War also affects the critical facilities and makes the situation worse as victims cannot get the vital basic services. According to media and unpublished reports, Tigrayan women have been victimized by gang rape and sexual violence. Furthermore, there is substantive evidence of intentional destruction and vandalization of health facilities due to the one-year-old-armed conflict. This study aimed to explore experiences of rape survivors in areas hit by armed conflict in the Tigray region of Ethiopia.

**Methods:**

In this qualitative study, a phenomenological study design was employed among Tigrayan sexual assault survivors in a war-ravaged of Tigray. Survivors were selected purposively and included in the study for an in-depth interview. An interview guide was used to collect the data. Audio records from the in-depth interviews in Tigrigna were transcribed verbatim and then translated into English for analysis. Atlas-ti 7 software was used to code the interview transcripts of the qualitative data, and categorizations and thematizing of the codes were done. Direct quotes were used to describe categories or themes.

**Results:**

Ten women who were survivors of sexual violence and rape related to the war in Tigray participated in the interview. The age of the women ranged between 16 and 30 years with a mean age of 21.7. Among the participants, five were teenagers, six were single and/or economically dependent on their family or husband, and two did not attend any school and were not able to read and write. This study has generated five major thematic areas: (1) infliction of long-lasting trauma on children (2) effects of the rape (3) means of escaping from rape and killings (4) home remedies as means of life saving in war affected areas, and (5) beyond rape.

**Conclusions:**

Rape in war-stricken Tigray has been widespread which includes teenagers and it caused immense psychological and physical damage to the survivors and their families. Damage of critical facilities such as the absence of safe houses for survivors and health services was an added complexity to the victims of rape. Hence, a coordinated effort by the government of Tigray and international partners is required to heal, support and rehabilitate the victims and rebuild the damaged health institutions and reequip the health facilities.

## Introduction

After the widespread use of rape and sexual violence during the genocide in Rwanda and ethnic cleansing in the former republic of Yugoslavia, the use of rape and sexual violence as weapon of war was considered as a human right violation and treated as a crime against humanity [1, 2]. Since the international criminal tribunal for Yugoslavia and Rwanda in 1993 and 1994 respectively, the United Nations Security Council has adopted Resolution 1325 to address the gender specific impacts of war and conflict on women and resolution 1820 on sexual violence as a weapon of war [3, 4].

In many places throughout the globe, rape has been used to “terrorize, torture, humiliate, and exterminate the ethnic “Other”” [5]. For instance, sever forms of rape and sexual violence was committed in Rwanda, Yugoslavia [5, 6], South Sudan, Nigeria [5], and the Democratic Republic of Congo (DRC) [7]. In the background of conflicts and civil war, sexual violence and rape in these places are ethnically and religiously motivated.

Current literature demonstrates that rape and sexual violence during war time are not the result of the war but it is commonly planned and targeted by design and policy [2]. ‘Rape as a weapon of war’ thus refers to sexual violence as having a systematic, pervasive, or officially orchestrated aspect, emphasizing that rapes ‘‘are not random acts, but carried out as deliberate policy” to terrorize and humiliate the victims [8]. There are well documented evidence of the use of mass rape, and sexual violence to advance military and political goals in Rwanda, Bosnia and other countries such as the DRC to intimidate, sabotage, punish or humiliate the community and enemies [2, 7, 9]. Despite an old misconceptions of rape as a byproduct of war, “rape, however, is used as a weapon of war to terrorize, torture, humiliate, and exterminate the ethnic “Other””[5].

Rape and sexual violence results in health, physical, psychological, social and societal impacts [10]. For instance, these days some direct health impact of the rape and sexual violence include infections by HIV and other sexually transmittable diseases, unwanted pregnancy and Fistula [11–13]. Furthermore, rape victims suffer from mental health problems caused due to stigma and discrimination [14, 15].

Following the eruption of the fighting between the Allied forces (the Ethiopian Defense forces, the special forces of the regions of Ethiopia, especially that of Amhara, and the Fano of Amhara, and Eritrean defense forces) and the Tigray defense forces (TDF) in November 2020, a mass rape and sexual violence, believed to be systematic in nature started in the Tigray region [16–18]; The level of severity and pervasiveness of the violence was reported to be high. For example, insertion of foreign bodies to the reproductive organs of Tigrayan women was reported by those who visited health institutions. For instance, between February and April 2021, 1,288 rape and sexual violence cases were reported in Tigray region [19]. The estimated number of women and girls raped during the conflict in the Tigray region of Ethiopia were reported to be more than 10,000 by Gesesew [19]. A damage assessment study that covered a wider area and longer duration of risk exposure (November to June) reported in 2022 [20] found about 120,000 rape victim girls and women. Under estimated reports can partially be explained as the Tigrayan women like their Afghan counterparts are reluctant to report their rape and sexual experience of violence due to fear of stigma and discrimination, cultural and societal value [21]. When it comes to the perpetrators, 44% and 33% of the victims have reported to have been raped by Ethiopian and Eritrean soldiers respectively, while the remaining 6% were raped by Amhara militia and 11% by unidentified perpetrators [19].

What makes the situation more complicated was that health institutions were targeted during the war. Infra-structures of the health institutions were damaged by shelling. They were looted and vandalized. Some compounds of the health facility and its rooms were occupied and fortified by troops [22]. Health professionals were fleeing due to security reasons or for being targeted. In some areas, health institutions gave services only during day time as six PM was the curfew time. Motor vehicles or cars including ambulances were not allowed to move, so emergency health services like post-pills, other family planning services, first-aid and medical services were not accessible to the needy ones. There is not any study, except one [19], which examined the nature of rape and sexual violence in the Tigray region of Ethiopia. Hence the aim of this study is to explore experiences of rape survivors in the war affected areas of Tigray region of Ethiopia.

## Theoretical framework

Feminist thoughts and activism have challenged the myth that rape is rare and exceptional highlighting that it is a common experience among girls and women [23, 24]. While liberal views of rape tend to consider rape as a gender neutral assault on individual autonomy, radical views of rape “contend that rape must be recognized and understood as an important pillar of patriarchy” [23, 25]. Feminist intellectuals made substantial contribution in terms documenting, investigating and raising consciousness about the problem of mass rape during wartime—rape during war like rape in peace time is identified not a crime to satisfy of sexual desire but derived by the motivation of a man to exert dominance over a woman [25–27]. In general, according to the feminist theory, “men may fight on different sides and for different reasons, in one sense they are all warriors on behalf of their gender—and the enemy is woman” [26, 28].

The other theory to explain rape during war time is the cultural pathology which has the character of psychoanalysis and look deep in to the countries cultural history [26]. There are many explanations as to why mass rape happens during war time according to the cultural pathology. The commonest explanation of the rape and sexual violence that happens in Tigray was related to military culture which fosters hostile attitudes towards women that commonly, culminate in feelings of entitlement to rape [26].

Currently, strategic rape theory is the most widely employed theory to inform mass wartime rape [26]. According to this theory, mass wartime rape represents just another form of ordinance similar to “bombs, bullets, or propaganda—that a military can use to accomplish its strategic objectives; rape is a tactic executed by soldiers in the service of larger strategic objectives” [26]. Even though supporters of this theory argue that military planner may not openly instruct their soldiers to rape, wartime rape is a coherent, coordinated, logical, and brutally effective means of prosecuting warfare [26].

The feminist, cultural pathology and strategic rape theories have all used to inform rape and sexual violence in Yugoslavia, Rwandan genocide, and other places with similar nature of war crime, genocide and ethnic cleansing [2, 29]. Similarly the three theories are gingerly mixed and used in a nested way to inform, guide the methodology and interpretation of the findings of this study. What makes their relevance valid in this particular context is that all have their own relevance in explaining some aspects of the nature of rape in Tigray war as the nature of the war has taken the posture of total war where every instrument of violence was used against all regardless of the distinction among combatants, civilians and protected entities. Besides reports of international organizations like Amnesty International and Human rights watch have witnessed massive atrocity crimes have been committed that gives similar context as the massive atrocity crimes including genocide in Yugoslavia and Rwanda. Thus, the relevance of the theories is in the context of Tigray is further reinforced by this similarity.

## Methods

### Context of the study area

Tigray is the northern most regional state of Ethiopia with a total population of around seven million (51.8% females); of the total population, 23.5% are females at their reproductive age [30]. It is bordered by Eritrea in the north, Amhara regional state in the South, Afar regional state in the East and Sudan in the west [30, 31]. Before the war in Tigray, there were two specialized hospitals, 38 hospitals, 224 health centers, and 741 health posts [32], and these numbers are now changed as most of the facilities are now damaged or out of function. About three-fourth of all the health facilities have been destroyed or vandalized [20].

### Design

Qualitative approach with phenomenological study design [33] was employed to dig out lived experiences among victims of rape due to the ongoing war in Tigray region since November 2020.

### Recruitment of study participants, sampling and information collection approach

War related sexual assault survivors were selected for the study from five zones of Tigray: eastern, south east, central, northwest and Mekelle. As a strategy to identify those sexual survivors related to the war that broke out in November 2020, we used local health office officials or district/local administrators. We planned to have at least two survivors from each zone with a minimum of ten study participants. In-depth interview (IDI) was used to collect the information in the month of May 2021 based on interview guide that was prepared based on previous experience and from literature. The interviews were done at households in places where the interviewees chose and felt comfortable to stay/sit and secured to talk after securing informed consent from each interviewee. Data collectors were university instructors and PhD students who can speak the local language and know the culture. When call was done to recruit data collectors, almost all of the applicants were males. The interviewers are public health professionals who are experienced in handling and dealing with GBV survivors. However, it is obvious that female experts of this caliber could make better interview. Victims might feel more comfortable with female interviewers. Thus, hiding sensitive information could be a limitation due to gender of the interviewers. To overcome these, training was given to the interviewers on the guide, privacy, confidentiality and on how to handle an interviewee’s reaction if any. Importantly, most of the survivors were informed to go to the health care centers or safe-house in the region to receive mental and psychological first aid and psycho-social support. Even some of them were being treated at the time of interview.

### Rigor and trustworthiness

Data collectors were recruited based on the previous experience on qualitative data collection and research. It conducted quality control checks to resolve discrepancies with the data. Debriefing was done among data collectors who were assigned in the same group. They were taking notes in addition to the sound recorder. Qualitative data collectors were communicating for follow up and for any problems that emerged during the interview.

### Data analysis

Verbatim audio recorded Tigrigna interviews were transcribed and then translated into English for analysis. Debriefing was conducted among the group members. Atlas-ti software was used to code the transcripts of the qualitative data; and categorizations and thematization of the codes were made. Direct quotes have been employed to describe the themes.

## Results

### Participants’ characteristics

In this study,  ten women who were survivors of rape and sexual violence related to the ongoing war in Tigray Regional State participated in the in-depth interview. Socio-demographic characteristics of the participants, obstetric and previous exposure to violence are depicted in Table 1. The mean age of the survivors was 21.7 (SD ± 5.23) years, with a range of 16–30 years; five of them were teen agers. Eight of the ten rape and sexual violence survivors have attended at least secondary school. Out of ten survivors, six of them were economically dependent either on their family member or partner. Regarding marital status, six of them were single; and two women become widowed because their husbands were killed by Eritrean defense forces during the rape and sexual violence scene. Three women while being gang raped were kept with the perpetrators for six hours to two weeks. Among the participants, none reported previous experience history of rape. Only one internally displaced woman was participated in the study.

**Table 1:** Socio-demographic and obstetric characteristics, history of rape, and displacement of women participants in Tigray, 2021

Code	Age	Marital Status	Educational Status	Occupation	Parity	History of Violence	Internally Displaced Person	Number of rapist
S1	20	Single	1st year university student	Student	0	None	No	1
S2	25	Married	10th grade	House wife	1	None	No	1
S3	17	Single	8th grade	Student	0	None	No	8
S4	18	Married	10th grade	House wife	0	None	No	1
S5	30	Single	Not attended any schooling	Merchant	1	None	Yes	1
S6	16	Single	Not attended any schooling	Daily Labourer	0	None	No	1
S7	18	Single	11th grade	Janitor	0	None	No	1
S8	19	Single	11th grade	Student	0	None	No	1
S9	24	Widowed	Diploma	Private bussiness	2	None	No	6
S10	30	Widowed	8th grade	Unemployed	2	None	No	4

N.B- Parity: is the number of times a woman has given birth to a live neonate (any gestation) or at 28 weeks or more, regardless of whether the child was viable or non-viable (i.e. stillbirths)

The findings of the study are summarized in five major thematic areas; long lasting trauma infliction on children, effects of the rape, means of escaping from rape and killings, home remedies as means of life saving in war affected areas, and beyond rape (Table 2).

**Table 2:** Showing how categories and themes were constructed

Codes	Catogarization	Themes
- Witnessed the overkillings of their family member - Son saw his mother being raped - Mother could not feed her neonate - Child crying because of separation from her mother by troop - Mother disappeared for weeks leaving children behind - No one looked after children	¬ Bad act exihibited to the significant others ¬ Deprivation of mother’s Care	Infliction of long lasting trauma on children
- Crying - Felt stress - Fear of exposing the secrete - Fear of stigma and discrimination - Taken forcefully & publicly - Could not move/ ambulation problem - Persistent pain, body sore - Bleeding - Broken teeth - First time experience - Being teenager - Life is over	¬ Psychological & Physical traumas ¬ Physiological change ¬ Body image disturbance ¬ Prefering teenagers ¬ Hunt for virginity	Effects of the rape
- To appear at the door at the first knock of the troop - Pretending to be having HIV infection - Disease condition - Courage - Being submisive	¬ Sacrificial for others ¬ Disease condition as protection ¬ Divine intervention ¬ Raped by HIV carrier for pretending to be carrier	Means of escaping from rape and killings
- Looting health institution - Destruction of physical structure - Presence of troops in the compound - Home remedies - Curfew	¬ Counselling ¬ Health service interruption ¬ Troop occupation ¬ Health service inaccessibility ¬ Alternatives to health care	Home remedies as means of life saving in war affected areas
- Blaming women for feeding Tigray defence force (TDF) - Accusing women of knowing where about of TDF forces - Hosting women in camps - Fisting - Uterine prolapse - Gang rape	¬ False accusation as means for & justification for rape ¬ Damaging reproductive organ	Beyond Rape

### Infliction of long lasting trauma on children

The length and severity of the effects of war depends on various conditions and become worest when it happens to children. The woman who is a mother of two and three years old daughters disappeared for weeks and left them with no one to looked after them. There was a similar report from another woman who had a son of thirteen years old. The son observed the act of rape. He was expelled out of the room after giving a strong slap on his face and made to stay in the dark compound alone. The mother witnessed her child urinate on his jeans after the slap. The mother also had a neonate/newly born baby and it was left unfed throughout the night. The mother failed to respond to the crying baby because she was trapped fiercely by a gang of Eritrean soldiers.

A child has been left crying in the street since the mother was taken by troops on the way back home from a market. The mother was dragged forcefully to a corner of the town where the soldiers have fortified. The crying child was looking for his mother for hours. The mother re-joined her son after being physically affected and unable to move properly and support the baby because of the gang rape; she was supported by a passerby and taken home along her child.

### Effects of the rape

A woman was taken forcefully by foreign troops to mean Eriterian defense forces to far farm yard for raping just after they killed her husband. There was an evidence of overkilling: the husband was shot dead on abdomen with first bullet and then on head with two bullets. The rape survivor women were crying when narrating their experience. For these women what happened a year ago is just yesterday. It always remains fresh and vivid in their immediate memory. Every moment of remembering is a painful live experience of re-living the excruciating experience. Fears of their secrete being exposed, and stigma and discrimination have been their daily nightmares that keeps them in fear and trembling more than the experience of victimization. Almost all women reported they had vaginal bleeding for days, weeks and for some of them for months. A woman reported that she felt alienated and detached from the society she once love and was proud of because no one came to her rescue when she was forcefully taken from a street in public and got raped. In fact, she knows that the rapist was threatening to those who wanted to rescue her. A woman reported that she has had persistent pain on her back which has resulted in difficulty to move. Similarly, another woman reported that she has been beaten by the group of rapist Eritrean soldiers and lost two of her teeth. She has been worried about her disfigured self-image.

*“In addition to being weakened and losing own business, supporting kids of two and three years as a single mother (in the absence of a husband) has become challenging for me*,” said a mother whose marriage was dissolved by this war. This war happening in Tigray region had been dismantling many families as reported by two survivors. Their households have become female headed houses and both women became widowed due to the current war; and they were in deep bereavement when they experienced the rape.

Safety is among the basic needs of human being in any situation. A women reported that half of the soldiers (four in number) were ravaging her dignty, safety and security. The rest were inspecting the surrounding areas to ensuring their safety by shooting at anyone appearing to the unfortunate scene even accidentaly. A teenage survivor who had sexual contact for the fist time said, *“I do no know why these troop were searching the young and virgine one?”* When enquired to explain further, she reasoned out that they needed safety to mean avoid infection and to satisfy their pleasure. The teenager said, “*I could not think of marriage or having a partner as I am the left over of rape,”* and went on saying, *“I could not also imagine continuing my educational career.”* A lady reported that her life could probably be complicated for she was totally dependent on her family. She mentioned that due to the first incident she failed to have HIV test because of doubting her aunt and later she forgot or no one to take her to the hospital rendering the diagnostic services. Still, she was waiting to take her. She regrets for not having the HIV test on time as it was fundamental for her safety. Though the Eriterian defense force harassed Tigrayan women indescriminately, the young and unmarried student survivors living under their families reported that their life is generally over because of the rape.

### Means of escaping from rape and killings

The Eritrean soldiers knocked door to door and killed many male youth. A young female she reacted and appread at the door for she was affraid that they would kill her brothers. One of the troops forced her to step out of her house. He forced her to lie inside the water reserviour used during rainy season.

As one lady reported, she told the soldiers that she was infected with HIV and appealed to let the rapist leave her, but they were not willing, rather they called their colleague who was HIV infected one to take match and he raped her. The rapists were very cruel and it was difficult to get rid of them. A similar experince from a lactating mother having reported that she repeatedly implored and begged the rapist: *“I am cardiac… please leave me… I have heart disease… I am advised by doctor not to have even another child… I just gave birth to this child at the gestational age of eight months”.* However, she could not escape from the hands of those cruel being what she prefered to call them ‘those Satans’.

Some of the participants were desperate to be rescued from such devastating situation by nothing else than the grace of God, divine intervention to end their fates. While others facing rape and dehumanization they exhibited that unique Tigrayan courage and defiance in the face of adversity at the pain of death and unbearable suffering, Tigrayans would destroy and evacuate the foreign troops to mean the Eritrean Defense Forces. The residents believed most crimes in their specific localities were committed by Eritrean soldiers. They proposed the current governing body to manage the issue, and they urged researchers and the elite to expose the crime to the international community and seeking support.

### Home remedies as means of life saving in war affected areas

During the armed conflict, government sectors and other private institutions were affected. The war has directly or indirectly affected health service delivery and/or its utilities. As a result of such inaccessibility, a woman could not prevent conception or get abortion service early. If at all a victims is to get abortion service, it will be too late because it requires travelling many kilometres from her town to a city.

To escape this hurdle, alternatives like home remedies and other options were practiced. A lady reported how she was treated at home with home remedies as health services was not accessible: *“… after two days of being raped and left thrown in a bush, I was seen by a child who told to his parent (father). After he understood my situation, the man picked and carried me to his home. I had bleeding, felt severe pain, I was panicking and startling, and my uterus was protruded outside of my sexual organ. I had been treated with steam and locally prepared herbals used by smocking to the injured body. I regained my conscious to some extent after twelve hours of stay. The pain churning my whole body minimized and later my uterus recoiled back and took its place. Then I was transported to the city where I could get further management….”*

### Beyond rape

In addition to the devastation on health systems and others, women reported that the act of the troops was beyond rape. The soldiers revealed immense hatred, and as part of the killings. They approached women with false accusation for being part of the war or various pretexts although the rapists’ intention was made by design and premeditated to abuse them sexually that they required not reasons at all. Most of the soldiers knocked the women’s doors, by accompanying accusing words such as ‘Junta’ (to mean the Tigrayan defence force). Despite this glaring fact, the soldiers accuse, the victims of appearing innocent but hiding in the houses their enemy that they are being actively involved in supporting the ‘Junta’. A woman was falsely accused of sending her brother to battle field. With this pretext, members of Eritrean Defence Force had taken her to their camp and kept her there for weeks. She had been given meagre amount of food only to stay alive so that she can be repeatedly gang raped rounds after rounds day in day out. She was kept as sex slave. During her ordeal begging them to stop and let her go home was a futile attempt. They used to reply: *“don’t you know why we have been sent here to Tigray? We came here to fight and rape Tigrayan women”.* Such vulgar languages, as the interviewees reported, were followed by insults and humiliation.

Similarly, another woman who was in post-partum period and was trying to persuade them to leave her for she had a baby and just gave birth recently as she was in immediate post-delivery time. However, they refused and raped her in group. Among the rapists, one was grating his teeth in anger and inflicting pain by inserting his fist into her vagina and saying they were there to make the Tigrayan uterus quit giving birth. Among the raped women, one reported that her uterus was prolapsed after a gang rape.

Besides, a woman reported that her personal property like mobile, money and necklace was rescued her from two abusers for they got their share. After they looted her property, they told their friends they left her to the four of their friends. Repeated reports about the Eritrean troops described humiliation and insults to the raped women. A lady reported that she was forced to have sex out of her culture and teasing on her thinking to have sex like human. He explained she should act like animals. Women reported that the level and types of abuse by the Eritrean troops were gang type of abuse, odd, and to the extent of nearly killing the women.

## Discussion

The same way Hutu men rape Tutsi women as a means to attack/destroy the Tutsi community, troops of Eritrean defence forces and its allies raped an estimated of about 120, 000 Tigrayan women.

The aim of this study was to explore experiences of sexual and gender-based violence experience among sexual abuse survivors in war affected areas of Tigray, Ethiopia. Violence negatively affects women’s physical and mental health and well-being. It also affects families [34]. Some of the family members have received physical abuses that were almost similar to the ones that have already happened to the rape victims. A mother reported that she is suffering from a double pain, the pain of both herself and her son. In this study, families were witnessing the scene of rape of their mother or family members and the over killings.

Children who were dependent on maternal care and support were deprived of the care by the troops. Mothers were caught by the gang rapists either in camps or abducted for the purpose of sexual abuse and children were left alone, where no one looked after them. Children were reacting to the act by crying but could not get a positive response from the gang rapists. In addition, some of the children were physically abused.

The victims were housewives who depend on the husbands for livelihoods; and some were students who were totally dependent on their families. This war was really disbanded many families, the socially valued institution. The family has been considered as the ultimate achievable goal by Tigrayans since every single adult, be it female or male are strictly and continuously advised by the community to have marriage and cherish family. Families that disintegrated because of the war ended up in emotional and economic crisis. The impact of female headed families on children would be immense as their moms are expected to cover the economic gap and household chores. What it makes worse is mothers could not regain normal emotional status; it would be difficult to accept without having justice, social healing or compensation.

In this study, gang rapes were reported and safety was an issue for rapists. Half of the troops engaged in ensuring safety and looking for any threatening from the opposition. Besides, teen- age survivors reported that teens were targeted for sexual abuse by the rapists for safety reasons to avoid infection and to satisfy their pleasure. These teen agers claimed such abuses would be extremely deter their future educational career and future life. As these segments of population are totally dependent, they definitely would face economic challenges. They fail to engage properly in education and economic careers owing to emotional, psychological or physical problems.

Women who were the sexual abuse survivors reported that they have been spending stressful days since the incidence. They have been suffering from memory of the situation, fear of getting their situation exposed. Almost all women reported either one or more of the health consequences of rape: vaginal bleeding, uterine prolapsed, persistent pain on back resulting in restriction of movements, physical injury, and loss of their physical structure resulting in self-image disturbances.

All women who were participated in this study attempted to escape from the experience of rape employing reasons like having bad health conditions, being in immediate post-delivery and other reasons. However, none of them escaped from the bad act, with the exception of one who reported to have reduced rapist because two of them got their own share in kind (her personal property) through looting. Gang rapes were done intentionally. This seems clear from similarity of the reasons given by the abusers, and the intention of the acts reported by Journalist of CNN. The intention was to cleanse the ethnicity of Tigrayans by the Amhara Fano. Its implication was to make the Tigrayan uterus out of use, by damaging the reproductive organs of Tigrayan women, and ultimately make them not being able to produce.

According to UNISDR, critical facilities are all the elements of infrastructure that supports essential services in a society like health institutions and its structure, electricity, water, and transport systems [35]. Tigrayans have faced all the deprivations for months, or even up to more than a year. Focusing on the health systems, physical structures of some of the health facilities were damaged by shelling, its tools were vandalized, health professionals fled. Due to various reasons like curfew and security problems, clients could not get the health services during war. It would be very difficult to get the health services from the few health facilities left from shelling because of several checkpoints, if travelling is a must. Women preferred to stay where they thought safe like home, without health services since they would face the same problem while travelling, according to the reports by the officials of the Regional Health Bureau of Tigray. Among the ladies who were visited the health facilities, one reported second experience of rape after having been trapped by the troops while travelling to get the health services for the first experience of rape. In addition, alternatives like home remedies and other options were practiced. A lady reported how she was treated at home with home remedies and rescued her life as health services was not accessible. Where there are no health services or utilization for months up to solid one year, one could imagine the extent of suffering by various segment of population, including mothers who need emergency health services, children, individuals who had health follow-up and others.

The troops approached women with pretexts although their intention was to abuse them sexually. With this pretext, gang troops raped Tigrayan women at their home in the presence of family members or at their barracks. Similarity of the gang’s practices and the reasons they verbalized, and the cruelty they exhibited have implied the execution of order from higher officials, thereby holding as a part of the strategy of war. The war seems well designed to destruct the functional smallest social unit called a family which is the nucleus of a society. If the mother of a family either psychologically or physically or both is affected the whole family will be affected; it will be worse on those who lost their husband or get divorced in relation to rape. As reported, the gang rape has been also intended to sterilize the womb of Tigrayan. If this is calculated in terms of how a woman who will not be having her children and descendants in five generation given a total fertility rate of four, she may lose 64 offspring of her blood line. According to the average of both estimated reports (more than 10,000 & about 100,000), 50,000 women experienced rape in Tigray, and this may result in Tigray not to have 3,200,000 Tigrayans due to rape related to war. It could suffice to explain, depending on the reports received by the participants, the level and types of abuse by the allied troops that were gang type, odd, systematic and deliberate and to the extent to killing the women.

It would not be easy like owning the Tigrayan cars which were looted and changing the plate number to convert/change the Tigrayan ethnicity to another by letting the vagina/uterine/ cervix bleed. The act has rather escalated courage and strong resistance, and condemnation of the acts that would be paid off for what they did. During the interview, the women were almost in areas under the control of the actors, and some of them on sustained severe pain and psychological trauma. As rape and its psychosocial problems is not an issue to be exposed to others for dialogue and ultimately look for support [34], it has little attention by rehabilitation programmes in order to establish the need for support and to develop appropriate community-based responses to these problems. Those women who experienced the violence and fear of other incidents of harassment have reported to be committed to fight the abusers.

The rape of Tigray is very unusual to explain only one or more theories per se. The act of blitz and mutual rape and accompanying atrocities were not even reported in Rwanda but in Darfur genocide that neither feminist theory nor liberal or cultural pathology theories are fully capable of explaining what happened to the women and girls of Tigray. The feminist assumption of male dominance explains part of the reality. Yet, it does not explain why the men of Tigray who are soldiers like the allied forces did not commit the same type of crime by design. The findings showed Tigrayan women and girls were raped not because of their feminine gender not for the satisfaction of the sexual urge of the rapists but because they were Tigrayans. Their identity has become *prima facia* evidence for being legitimate prey of rapists which contradicts to the assumption of feminists that all men are in solidarity and women are the enemy. Instead “men may fight on different sides and for different reasons, in one sense they are all warriors on behalf of their gender—and the enemy is woman”. Other than Tigrayan women not all women were systematically and by design raped. The liberal idea of rape that argues for a gender neutral explanation as an “assault on individual autonomy” is true to explain to the rape in Tigray in terms of individual level analysis that rape victims were devoid of personal honor, autonomy and dignity. However, given the pervasive nature, objective and consequence of rape limiting the nature and purpose of rape to the prohibition of personal autonomy in the case of Tigray is a total understatement of the reality, because rape in Tigray is meant to destroy Tigray as a society and as an entity.

The cultural pathology which has the character of psychoanalysis and look deep in to the countries cultural history and the culture of violence, dehumanization and hatred legitimizing rape and other atrocity crimes explains the big part of the phenomena of rape in Tigray. Nevertheless, new and sui generis types of crimes which have never been observed in Ethiopia, Eritrea or Somalia were committed against Tigray like mutual rape which does not have any dictionary meaning in their respective languages that makes the cultural pathology theory too narrow to explain everything of the phenomenology of rape in Tigray. The anthropological theory that explains in terms of soldiers using the body of women and girls as battle ground is also relevant in Tigray war though this was done only by the part of allied forces against Tigrayans.

The strategic rape theory that underscores the use and weaponization of rape as tool of war and part of strategic decision making in war is the best fit theoretical caveat that explains the nature of Tigray rape. However, this theory falls short of explaining the excess violence and brutality, the dramatization and ritualization of rape and over killing which cannot be explained achieving strategic objectives of war by weaponization of rape. Because the excesses had no tangible war objective to attain other than deep down hatred and the desire to exterminate the people of Tigray by killing, raping, separating families, destroying their cherished social and cultural values and institutions, and at last by hampering reproduction through the deliberate and forced sterilization of women by torturing the womb of Tigray.

Therefore, none of the above theoretical caveats are capacious enough to explain the true nature of rape in Tigray that indicates the imperative for coining net terminology that fits to the sui generis nature of the violence. As indicated above in the findings section, the actions, objectives, and declared intents as evidenced by the rapists’ repeated utterances perfectly fit the definition and elements of crimes of genocide as provided in the convention on the prevention and punishment of crimes of genocide and ICC’s guideline on elements of atrocity crimes. Therefore, it is apt to call the rape of Tigray in its proper name, genocidal rape.

## Conclusions

In the experience of the interviewed women and girls, sexually abused victims along with their families have been impacted psychosocially and physically and deprived of the health services due to war which seem to be systematic and deliberate. Since many of the basic services have been stopped in the area, seeking the support of the international agencies could be mandatory. Adaption of the experience of other countries to support the survivors is crucial [36].

## Data Availability

The datasets used and/or analysed during the current study are available from the corresponding author on reasonable request.
